# Modeling *Clostridium difficile* in a hospital setting: control and admissions of colonized and symptomatic patients

**DOI:** 10.1186/s12976-019-0098-0

**Published:** 2019-01-31

**Authors:** Farida Chamchod, Prasit Palittapongarnpim

**Affiliations:** 1 Department of Mathematics, Faculty of Science, Mahidol University, Bangkok, Thailand; 2 Department of Microbiology, Faculty of Science, Mahidol University, Bangkok, Thailand

**Keywords:** Transmission of *Clostridium difficile*, Admission of patients, Control implementation, Disruption of the gut flora

## Abstract

**Background:**

*Clostridium difficile* (*C. difficile*) infection is an important cause of healthcare-associated diarrhea. Several factors such as admission of colonized patients, levels of serum antibodies in patients, and control strategies may involve in determining the prevalence and the persistence of *C. difficile* in a hospital unit.

**Methods:**

We develop mathematical models based on deterministic and stochastic frameworks to investigate the effects of control strategies for colonized and symptomatic patients and admissions of colonized and symptomatic patients on the prevalence and the persistence of *C. difficile*.

**Results:**

Our findings suggest that control strategies and admissions of colonized and symptomatic patients play important roles in determining the prevalence and the persistence of *C. difficile*. Improving control of *C. difficile* in colonized and symptomatic patients may generally help reduce the prevalence and the persistence of *C. difficile*. However, if admission rates of colonized and symptomatic patients are high, the prevalence of *C. difficile* may remain high in a patient population even though strict control policies are applied.

**Conclusion:**

Control strategies and admissions of colonized and symptomatic patients are important determinants of the prevalence and the persistence of *C. difficile*.

## Background

*Clostridium difficile* (*C. difficile*) which is a spore-forming gram-positive bacillus is a causative agent of *C. difficile* infection (CDI). It has become a leading cause of healthcare-associated diarrhea resulting in morbidity, mortality, and hospitalized costs to patients and healthcare institutions in many countries [1]. For example, in the United States, it was estimated that *C. difficile* may be responsible for 333,000 cases per year costing approximately $3.2 billion and causing 15,000–20,000 deaths [2]. The clinical spectrum of CDI ranges from asymptomatic colonization to self-limited mild diarrhea, severe diarrhea, life-threatening disease such as toxic megacolon and sepsis, and death [1, 3]. Although reported cases of CDI have been declining in the recent years, the incidence rates still surpass infection rates of methicillin-resistant *Staphylococcus aureus* in several areas of the United States and Europe [4–6].

*C. difficile* resides in the normal intestinal microbiota of 1–3% healthy adults and generally most colonized people with the normal gut flora remain asymptomatic [2]. However, when the normal gut flora of patients is disrupted to conditions that favor proliferation of *C. difficile*, those who are exposed to *C. difficile* spores or those who are already asymptomatically colonized may develop CDI [7, 8]. It has been well recognized that antimicrobial exposure is an important risk factor linked to alterations of the gut flora and the development of CDI [3]. Nearly every antimicrobial can lead to alteration and infection; however, broad-spectrum agents such as clindamycin, cephalosporins, and fluroquinolones are most frequently reported causes [1, 9]. Note that despite the considerably lower rate in comparison to hospitalized patients, low-risk populations such as individuals with no recent health-care histories, pregnant women, and children in a community setting can also develop CDI [10, 11].

Another factor that plays a crucial role on the development of CDI is host immune responses. As pathogenic effects of *C. difficile* are typically exerted through the production of toxin A and toxin B, patients who have high levels of serum immunoglobulin G (IgG) and A (IgA) against *C. difficile* toxins are normally protected from diarrhea and hence remain asymptomatic [12, 13]. On the other hand, patients who have low levels of serum antibodies are more likely to develop clinical symptoms. In addition, the high levels of serum antibodies may also protect patients from recurrence of CDI [13].

Elimination of CDI requires restoration of gut flora and patients with mild disease can occasionally be treated by ceasing antimicrobial therapy. Metronidazole and vancomycin are first-line therapeutic agents for treating mild and severe CDI, respectively [1]. Both are effective with 95–100% response rates for mild disease but the former is less efficacious than the latter for severe disease [14].

Mathematical models have been used to investigate transmission dynamics of *C. difficile* but they are still not numerous [15–21]. Starr et al. [15] developed a stochastic model based on the herd immunity hypothesis of CDI outbreaks to understand *C. difficile* epidemiology. To determine most important factors influencing transmission of *C. difficile*, Lanzas et al. [16] proposed a mathematical model for patients with and without protection against CDI and underlined an important role of colonization at admission. Rubin et al. [17] designed an agent-based simulation model to explore six interventions on transmission of *C. difficile* and suggested improved hand hygiene compliance and isolation practices for suspected C. difficile cases as effective ways to control the spread of *Clostridium difficile*. Yakob et al. [18, 19] constructed mathematical models to investigate the efficacy of control measures, and mechanisms of hypervirulent and endemic strains of *C. difficile*. Codella et al. [20] developed an agent-based simulation model for investigating infection control and estimating transition probabilities. Recently, van Kleef et al. [21] constructed an individual-based transmission model incorporated with data of patient movements and found that vaccination may be most desirable in groups of patients who take high broad-spectrum antimicrobial agents.

In this present study, we develop mathematical models to investigate the effects of additional control measures targeted at colonized and infected patients (e.g. isolation or cohort nursing) and admissions of colonized patients on the prevalence of *C. difficile* and the tendency of infection cases to be temporarily driven out. Although the emergence of CDI among low-risk populations has been increasingly reported, it has been suggested in several studies that more than 90% of hospitalized patients who develop CDI have recent antimicrobial exposure [22, 23]. Hence, in this work, instead of considering two groups of patients who take and do not take antimicrobial agents, we only focus on the former group of patients who are at higher risk and promptly affected. Our aims are 1) to investigate how additional controls targeted at colonized and infected patients and admissions of colonized patients affect the transmission dynamics of *C. difficile* among patients who receive antimicrobial agents and have disruption of the gut flora and the tendency of *C. difficile* infection to be temporarily driven out, and 2) to identify factors that may have a significant impact on the prevalence and the persistence of *C. difficile*.

## Methods

To describe the transmission dynamics of *C. difficile* among patients with antimicrobial exposure in a hospital unit, patients are divided into four categories: uncolonized patients (*U*), colonized patients with high levels of serum antibodies (*A*), colonized patients with low levels of serum antibodies (*C*), and symptomatic patients (*I*). Uncolonized patients are those who currently have the disruption of gut flora from antimicrobial exposure but have not yet been colonized by *C. difficile*. Colonized patients with high levels of serum antibodies generally remain asymptomatic and do not develop severe clinical symptoms. For those who have low levels of serum antibodies and are colonized by *C. difficile*, they either leave the hospital or develop clinical symptoms. The total number of inpatients in the hospital, *N*=*U*+*A*+*C*+*I*, is kept constant in this study to reflect the fixed number of beds and the dynamics of admissions and discharges of inpatients.

It is assumed that admissions of patients are at rate *Λ* per day with the probabilities *λ*_*A*_,*λ*_*C*_, and *λ*_*I*_ of patients having high levels of serum antibodies and colonized, having low levels of serum antibodies and colonized, and having clinical symptoms, respectively. Those quantities usually vary in many hospital settings. Based on some preceding studies, the prevalence of colonized patients with toxigenic strains of *C. difficile* at admission is approximately 10% [24]. It was estimated that 60% of healthy people have high levels of serum antibodies, IgG and IgA, to *C. difficile* even if *C. difficile* colonization is absent (*p*=0.6, where *p* denotes the probability of having high levels of serum antibodies) [13, 25]. We thereby assume that *λ*_*A*_=0.06 and *λ*_*C*_=0.04. Some previous studies also suggested that approximately 1% of admitted patients are symptomatic at admission or otherwise develop clinical symptoms within 3 days after admission [26, 27]. Consequently, we assume *λ*_*I*_ to be 0.01 in our study.

In the model, discharges of patients occur at rates of *γ*_*U*_,*γ*_*A*_, and *γ*_*C*_ for patients who are uncolonized, colonized with high levels of serum antibodies, and colonized with low levels of serum antibodies, respectively. For simplicity, discharge rates of patients who are not symptomatic are set to be 1/5 per day, which is the inverse of the average length of stay of patients in a hospital [28]. Moreover, such length is approximately equal to the average length that colonized patients with low levels of serum antibodies become symptomatic [29]. Hence, we assume that after 5 days colinized patients who have low levels of serum antibodies either become symptomatic with the probability *q* or leave the hospital unit with the probability 1−*q*. Normally, *q* ranges from 10 to 40% [27, 30–32]. In this study, *q* is approximated to be 20% [30]. For symptomatic patients, we assume that all must be treated at rate of *ν* where 1/*ν* is the average length of a treatment course and it is assumed to be 10 days [8, 33]. Mortality from CDI varies among settings and depends on the virulence of strains. It is approximated to occur in 10% of the symptomatic patients in the model [2, 22]. Moreover, in the model, patients may be unsuccessfully treated and die due to CDI with the probability *r*. After successful treatment, treated patients are assumed to be colonized with *C. difficile* again. By this assumption, the model allows recurrence to occur in some treated patients with the same probability *q* with the probability of developing symptoms.

Transmission of *C. difficile* among patients is simply assumed to be density-dependent since patients normally shed a large number of spores in their stool that contaminate their hands, environmental surfaces, equipment, and hands of health care workers. This assumption is in agreement with some preceding modeling studies [16]. Because symptomatic patients heavily contaminate their environment in comparison to colonized patients, it is assumed in this study that *β*_*I*_>*β*_*A*_=*β*_*C*_. According to the infection control guidelines, symptomatic patients should be isolated in single rooms with private toilet facilities to a feasible extent and contact precautions should be applied to limit the spread of *C. difficile* [1]. However, it is quite often in many hospitals that control practices do not meet the guidelines [2]. Hence, *σ* is incorporated in the model to reflect additional control practices targeted at symptomatic patients. There are no additional attempts if *σ*=1. The attempts fully prevent transmission of *C. difficile* from symptomatic patients if *σ*=0. Similarly, *ε* is incorporated to reflect additional control practices applied to colonized patients.

**Fig. 1:**
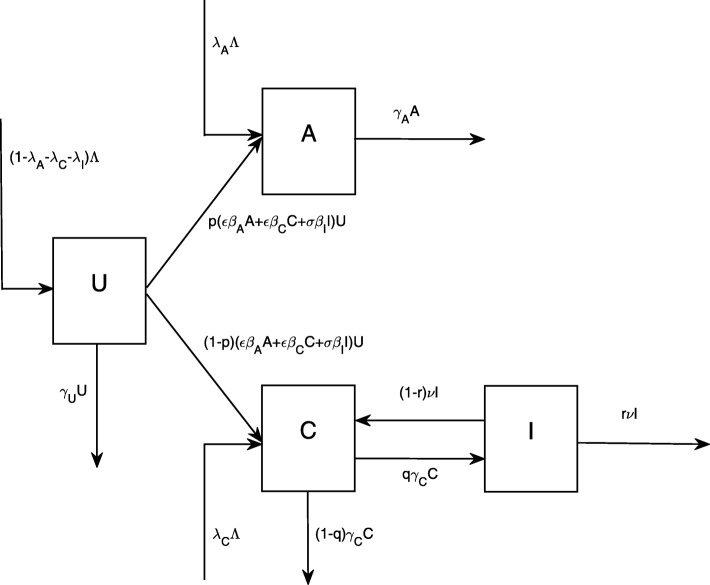
Flow diagram. Flow diagram for describing transmission dynamics of *C. difficile* among patients who have disruption of the gut flora in a hospital setting. Patients are categorized into four groups: uncolonized (*U*), colonized with high levels of serum antibodies (*A*), colonized with low levels of serum antibodies (*C*), and symptomatic (*I*)

From the aforementioned assumptions, the transmission dynamics of *C. difficile* among patients who have antimicrobial exposure in the hospital can be described by

1 $$ \begin{aligned} \frac{dU}{dt} & = (1-\lambda_{A}-\lambda_{C}-\lambda_{I})\Lambda -(\epsilon\beta_{A}A+\epsilon\beta_{C}C+\sigma\beta_{I}I)U-\gamma_{U}U \\ \frac{dA}{dt} & = \lambda_{A}\Lambda+p(\epsilon\beta_{A}A+\epsilon\beta_{C}C+\sigma\beta_{I}I)U-\gamma_{A}A\\ \frac{dC}{dt} & = \lambda_{C}\Lambda+(1-p)(\epsilon\beta_{A}A+\epsilon\beta_{C}C+\sigma\beta_{I}I)U+(1-r)\nu I-\gamma_{C}C\\ \frac{dI}{dt} & = \lambda_{I}\Lambda+q\gamma_{C}C-\nu I \end{aligned}  $$

with *Λ*=*γ*_*U*_*U*+*γ*_*A*_*A*+(1−*q*)*γ*_*C*_*C*+*rνI*. Note that *Λ* depends on the dynamics of inpatients and treatment rate of symptomatic patients. The parameters used in the model are shown in Table 1 and the flow diagram for movements between compartments of inpatients is illustrated in Fig. 1. The deterministic model captures dynamics of inpatients by considering patient admissions and discharges, immune responses that prevent symptomatic cases, the development of clinical symptoms, and treatment rate. The model also takes into account additional control measures applied to colonized and symptomatic patients to prevent further transmission from colonized and symptomatic patients to uncolonized patients.

**Table 1:** A list of parameters for *Clostridium difficile*

Description	Symbol	Sample value	References
Probability of having high levels of serum antibodies to *C. difficile*	*p*	0.6	[13, 25]
Probability of colonization at admission with high levels of serum antibodies	*λ*_*A*_	0.06	[24, 25]
Probability of colonization at admission with low levels of serum antibodies	*λ*_*C*_	0.04	[24, 25]
Probability of having symptomatic patients at admission	*λ*_*I*_	0.01	[26, 27]
Discharge rate of uncolonized and colonized patients (day ^−1^)	*γ*_*U*_,*γ*_*A*_,*γ*_*C*_	0.2	[28]
Probability of developing clinical symptoms	*q*	0.2	[30]
Probability of death due to *C. difficile*	*r*	0.1	[2, 22]
Treatment rate (day ^−1^)	*ν*	0.1	[8, 33]
Transmission rate from colonized patients	*β*_*A*_,*β*_*C*_	0.006	[2, 16, 29]
Transmission rate from symptomatic patients	*β*_*I*_	0.008	[16, 29]
Control effort factor for symptomatic patients	*σ*	0−1(1,0.1)	(varying)
Control effort factor for colonized patients	*ε*	0−1(1,0.1)	(varying)

When only a small unit in a hospital (e.g. an intensive care unit) is explored for transmission dynamics of *C. difficile*, the total number of patients can become very small. In such case, although the deterministic model (1) can be used to describe the mean results of its stochastic version, it may not be able to capture the possibilities of having local extinctions and reemergence of *C. difficile* among patients in the unit and how certain parameters affect those events. Hence, in this study, a continuous-time Markov chain (CTMC) model is developed and studied alongside the deterministic model. In the CTMC model, state variables are discrete and satisfy

$$S(t), A(t), C(t), I(t)\in\{0,1,2,\ldots, N\}, $$

where *t*∈ [0,*T*). Due to the fixed number of beds, the number of susceptible patients can be obtained from the equation *S*(*t*)=*N*−*A*(*t*)−*C*(*t*)−*I*(*t*). The transition probabilities associated with the stochastic process are defined for a small period of time *δt*>0 as follows:

$$\begin{aligned} p_{(a,c,i),(a+j,c+k,i+l)}(\delta t) & = P((A(t+\delta t), C(t+\delta t), I(t+\delta t))=(a+k,c+j,i+l)|\\ & (A(t),C(t),I(t))=(a,c,i)), \end{aligned} $$

where *a*,*c*, and *i* are the values of discrete random variables. The probabilities for describing patient transitions according to changes of disease states and demographic variability are shown in Table 2. The model is solved by the Gillespie algorithm for certain sample paths of state variables [34, 35], cumulative time in the absence of colonized and symptomatic patients, and the number of absence periods of each patient group in the hospital unit.

**Table 2:** Possible changes in the stochastic model

Event	Transition	Probability of a transition event
Admission of a colonized patient with high antibody levels	*A*→*A*+1	*λ*_*A*_ *Λ* *δ* *t*
Colonization in a patient with high antibody levels	*A*→*A*+1	*p*[*ε*(*β*_*A*_*A*+*β*_*C*_*C*)+*σβ*_*I*_*I*]*δt*
Discharge of a colonized patient with high antibody levels	*A*→*A*−1	*γ*_*A*_ *A* *δ* *t*
Admission of a colonized patient with low antibody levels	*C*→*C*+1	*λ*_*C*_ *Λ* *δ* *t*
Colonization in a patient with low antibody levels	*C*→*C*+1	(1−*p*)[*ε*(*β*_*A*_*A*+*β*_*C*_*C*)+*σβ*_*I*_*I*]*δt*
Colonization in a patient who is successfully treated	*C*→*C*+1	(1−*r*)*νIδt*
Discharge of a colonized patient with low antibodies levels	*C*→*C*−1	*γ*_*C*_ *C* *δ* *t*
Admission of an infectious patient	*I*→*I*+1	*λ*_*I*_ *Λ* *δ* *t*
Infection of in colonized patient	*I*→*I*+1	*q* *γ*_*C*_ *C* *δ* *t*
Recovery or death from an infection	*I*→*I*−1	(1−*r*)*νIδt*

## Results

### The basic reproduction number (*R*_0_)

When there is no admission of colonized and symptomatic patients (*λ*_*A*_=*λ*_*C*_=*λ*_*I*_=0), the basic reproduction number can be calculated by using the method of next-generation matrices [36, 37]. Deriving two matrices, **F** and **V**, from the Jacobian matrices of the $\mathcal {F}$-matrix of new infections and the $\mathcal {V}$-matrix of compartmental movements at the disease-free steady state ((*U*,*A*,*C*,*I*)=(*N*,0,0,0)) gives:

$$\mathbf{F}=\left[ \begin{array}{ccc} p\epsilon\beta_{A}N & p\epsilon\beta_{C}N & p\sigma\beta_{I}N\\ (1-p)\epsilon\beta_{A}N & (1-p)\epsilon\beta_{C}N & (1-p)\sigma\beta_{I}N\\ 0 & 0 & 0 \end{array}\right], \; \text{and } \; \mathbf{V}=\left[ \begin{array}{ccc} \gamma_{A} & 0 & 0 \\ 0 & \gamma_{C} & -(1-r)\nu\\ 0 & -q\gamma_{C} & \nu \end{array}\right]. $$

The basic reproduction number is defined as the spectral radius of **FV**^−1^ as follows:

$$R_{0}=\frac{p\epsilon\beta_{A}N}{\gamma_{A}}+\frac{(1-p)\epsilon\beta_{C}N}{\gamma_{C}}\frac{\nu}{(1-q(1-r))}+ \frac{(1-p)\sigma\beta_{I}N}{\nu}\frac{q}{(1-q(1-r))}. $$

Hence, *C. difficile* cannot persist in the hospital when there are no admissions of colonized and symptomatic patients if *R*_0_<1 and it is prevalent if *R*_0_>1. Since admissions of colonized or symptomatic patients often occur, we then explore the following case *λ*_*A*_≠0, *λ*_*C*_≠0, and *λ*_*I*_≠0. In such case, the disease-present steady state only exists or *C. difficile* always persists in the patient population. However, due to several nonlinear terms in the model, analytic results are not obtainable. Hence, we investigate the effects of additional control measures targeted at colonized and symptomatic patients and admissions of colonized and symptomatic patients numerically in both deterministic and stochastic frameworks.

### Deterministic results

**Fig. 2:**
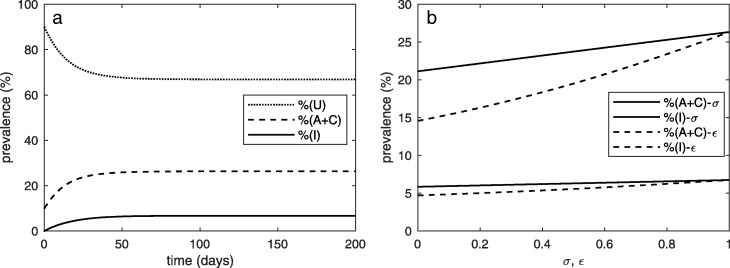
Prevalence. **a** The prevalence of *C. difficile* among patients in a hospital unit. **b** Effects of control factors for colonized and symptomatic patients (*ε*&*σ*, respectively) on the prevalence of *C. difficile*. When *σ* varies, *ε* is fixed. In a similar way, when *ε* varies, *σ* is fixed

According to some previous studies, the prevalence of *C. difficile* colonization in a hospital unit is approximately 10–25% [38]. Figure 2a shows our baseline results for the prevalence of patients in each disease category for *N*=20. From our parameter setting, the prevalence of colonized and symptomatic patients remains at a lower level as compared to uncolonized patients. Without additional control measures applied to colonized and symptomatic patients (*σ*=*ε*=1,*R*_0_=0.57), the prevalence of colonized patients is approximately 26% and the prevalence of symptomatic patients is approximately 7%. Consequently, the acquisition rate of *C. difficile* during an admission is approximately 16% in our work. By varying the control factors (0≤*σ*≤1,0≤*ε*≤1) as shown in Fig. 2b, it can be clearly seen that additional control measures targeted at colonized and symptomatic patients may have a greater impact on the prevalence of colonized patients as compared to symptomatic patients. With more stringent approaches applied to colonized and symptomatic patients, the prevalence of *C. difficile* is reduced. Moreover, applying additional control measures to colonized patients may help reduce the prevalence of *C. difficile* than applying additional control measures to symptomatic patients.

**Fig. 3:**
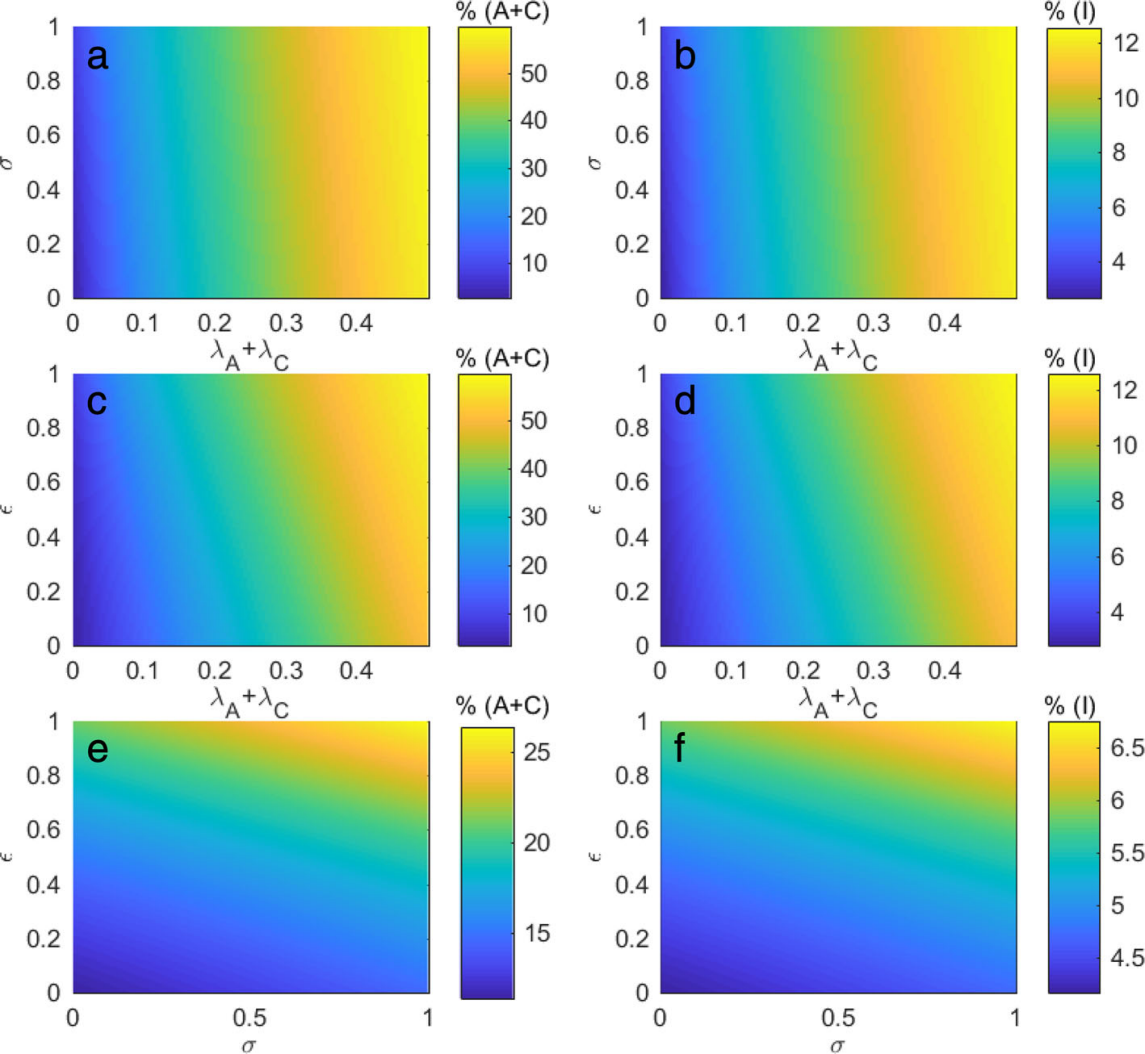
Control effort and admission of patients. **a**–**b** Effects of the control factor for symptomatic patients and the probability of patients being colonized at admission on the prevalence of colonized and symptomatic patients, respectively. **c**–**d** Effects of the control factor for colonized patients and the probability of patients being colonized at admission on the prevalence of colonized and symptomatic patients, respectively. **e**–**f** Effects of control factors for colonized and symptomatic patients on the prevalence of colonized and symptomatic patients

Figure 3a and b show the prevalence of colonized and symptomatic patients when the probability of patients being colonized with *C. difficile* at admission and the control factor for symptomatic patients vary. Our results suggest that changes in the probability of patients being colonized at admission have a more dramatic impact on the prevalence of colonized and symptomatic patients than changes in the control factor for symptomatic patients. Even though more strict control methods are applied to symptomatic patients (*σ*→0), the prevalence of colonized and symptomatic patients is still not significantly reduced if large numbers of colonized patients are admitted. Similarly, Fig. 3c and d demonstrate that the prevalence of colonized and symptomatic patients increases significantly according to the higher probability of patients being colonized at admission as compared to the control factor for colonized patients. Although more stringent control methods are applied to colonized patients, the prevalence of colonized and symptomatic patients is not considerably decreased. In addition, Fig. 3e and f demonstrate the effect of control factors on the prevalence of colonized and symptomatic patients when the probability of patients being colonized at admission is fixed. Our results suggest that the lower the control factors, the lower the prevalence of *C. difficile* among patients. Equivalently, improving control policies towards colonized and symptomatic patients may help reduce the prevalence of *C. difficile* among patients. Moreover, changes in the control factor for colonized patients may have a greater impact on the prevalence of colonized and symptomatic patients than changes in the control factor for symptomatic patients. Consequently, our results suggest improving control policies for colonized patients.

### Stochastic results

**Fig. 4:**
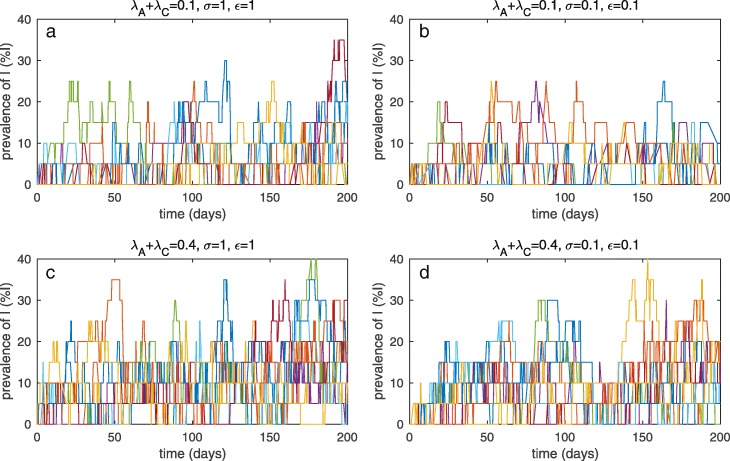
Stochastic results for *N*=20 during 200 days (20 realizations). Prevalence of symptomatic patients in a hospital unit (*N* = 20): (A) *λ*_*A*_ + *λ*_*C*_ = 0.1,*σ* = 1,*ε* = 1, (B) *λ*_*A*_ + *λ*_*C*_ = 0.1,*σ* = 0.1,*ε* = 0.1, (C) *λ*_*A*_ + *λ*_*C*_ = 0.4,*σ* = 1,*ε* = 1, and (D) *λ*_*A*_+*λ*_*C*_=0.4,*σ*=0.1,*ε*=0.1

When the total number of patients is small (*N*=20 patients in this study), random effects may have an impact on local extinctions and reemergence of *C. difficile* among patients. In this work, admissions of colonized and symptomatic patients are present in the model. Hence, even though *C. difficile* becomes extinct, it can shortly reemerge again in the patient population as a consequence of new admissions of colonized or symptomatic patients (see Fig. 4a–d). Figure 4a–d show that the prevalence of symptomatic patients has a tendency to decrease and symptomatic patients are more likely to be absent in the hospital unit when more stringent control policies are applied to colonized and symptomatic patients (*σ*→0,*ε*→0). Note that 1) only 20 realizations of stochastic results are shown here while 800 realizations are used for calculating the average quantities associated with stochastic results, and 2) our stochastic results are simulated for 200 days. We further investigate the cumulative absent time for each group of patients in a hospital and the number of absences of each group of patients (see Table 3). The cumulative absent time for each patient group is calculated from the average total time that any patients in each disease category are not present in the hospital unit (when *A*=0,*C*=0, or *I*=0) of 800 realizations and the absent number is calculated from the average number of absences of colonized or symptomatic patients on the time domain. Hence, the former term reflects how long colonization or infection is absent in the hospital and the latter term reflects how often patients in each group are not present in the hospital. When stringent control approaches are applied, the cumulative absent time for each disease category and the corresponding absent number increase. For the absence of symptomatic patients, due to the much longer absent time, the absent number remains the same in the results. Our results in Table 3 also demonstrate that applying stringent control policies to colonized patients may result in longer cumulative absent time of colonization and infection and higher absent number. These results are in agreement with the deterministic results.

**Table 3:** Cumulative absent time and absent number of patients in each disease group when the total number of patients in a hospital unit is small (*N*=20)

Admission and control factors	Cumulative absent time in percent	Absent number
*λ*_*A*_+*λ*_*C*_=0.1,*σ*=1,*ε*=1	*%*(*t*_*A*_,*t*_*C*_,*t*_*I*_)=(7.54,11.63,34.60)	(*N*_*A*_,*N*_*C*_,*N*_*I*_)=(9,10,7)
*λ*_*A*_+*λ*_*C*_=0.1,*σ*=0.5,*ε*=1	*%*(*t*_*A*_,*t*_*C*_,*t*_*I*_)=(9.07,13.08,35.57)	(*N*_*A*_,*N*_*C*_,*N*_*I*_)=(9,10,7)
*λ*_*A*_+*λ*_*C*_=0.1,*σ*=0.1,*ε*=1	*%*(*t*_*A*_,*t*_*C*_,*t*_*I*_)=(10.93,14.09,37.22)	(*N*_*A*_,*N*_*C*_,*N*_*I*_)=(10,12,7)
*λ*_*A*_+*λ*_*C*_=0.1,*σ*=1,*ε*=0.5	*%*(*t*_*A*_,*t*_*C*_,*t*_*I*_)=(10.82,16.63,38.52)	(*N*_*A*_,*N*_*C*_,*N*_*I*_)=(11,11,7)
*λ*_*A*_+*λ*_*C*_=0.1,*σ*=1,*ε*=0.1	*%*(*t*_*A*_,*t*_*C*_,*t*_*I*_)=(14.75,21.28,42.32)	(*N*_*A*_,*N*_*C*_,*N*_*I*_)=(13,13,7)
*λ*_*A*_+*λ*_*C*_=0.1,*σ*=0.1,*ε*=0.1	*%*(*t*_*A*_,*t*_*C*_,*t*_*I*_)=(19.52,23.91,44.21)	(*N*_*A*_,*N*_*C*_,*N*_*I*_)=(14,14,7)

Average results are obtained during 200 days from 800 realizations

**Fig. 5:**
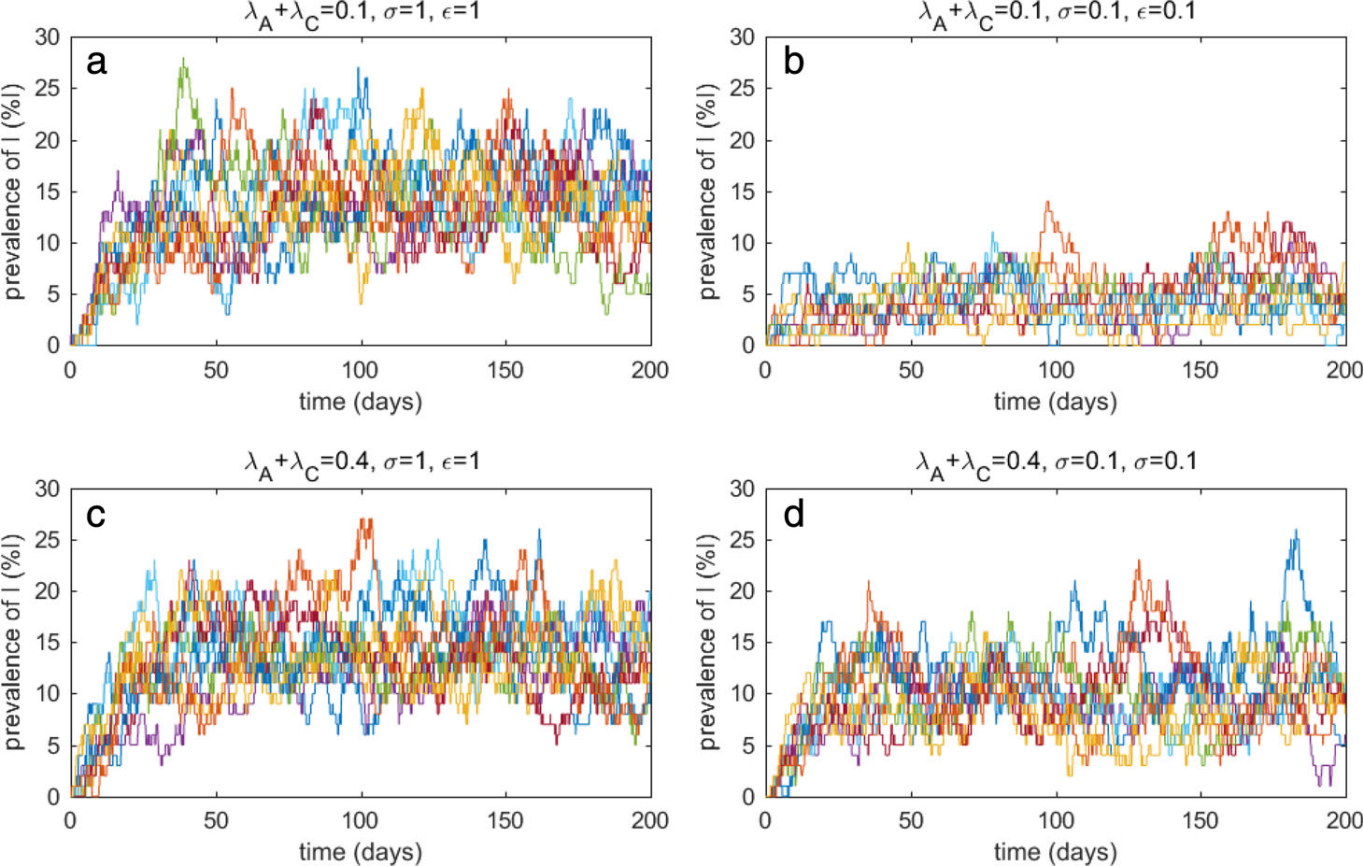
Stochastic results for *N*=100 during 200 days (20 realizations). Prevalence of symptomatic patients in a hospital unit (*N* = 100): **a** *λ*_*A*_ + *λ*_*C*_ = 0.1,*σ* = 1,*ε* = 1, **b** *λ*_*A*_ + *λ*_*C*_ = 0.1,*σ* = 0.1,*ε* = 0.1, **c** *λ*_*A*_ + *λ*_*C*_ = 0.4,*σ* = 1,*ε* = 1, and **d** *λ*_*A*_+*λ*_*C*_=0.4,*σ*=0.1,*ε*=0.1

In Fig. 5a–d, when the total number of patients is large (*N*=100 patients), stochastic effects have a smaller impact on temporary extinctions of *C. difficile* among patients and hence local extinctions of *C. difficile* rarely occur. The prevalence of symptomatic patients is reduced when more stringent control measures for colonized and symptomatic patients are applied (*σ*→0,*ε*→0). However, if very large proportions of colonized patients are admitted, the prevalence of symptomatic patients still remains high even though additional control measures are applied to colonized and symptomatic patients.

## Conclusions and discussion

To investigate transmission dynamics of *C. difficile* among patients with disruption of the gut flora in a hospital setting, we develop mathematical models based on deterministic and stochastic frameworks. The models are employed to explore the effects of additional control measures for colonized and symptomatic patients and admissions of colonized and symptomatic patients on the prevalence and the persistence of *C. difficile*.

The basic reproduction number (*R*_0_) is calculated when there are no admissions of colonized and symptomatic patients. The quantity suggests that the prevalence of *C. difficile* depends on several factors such as transmission rate, control strategies, the proportion of colonized patients who have high or low levels of serum antibodies, discharge rates, etc. For example, improving control policies for symptomatic patients results in decreased values of *σ* and *R*_0_ and that can lead to the lower prevalence of *C. difficile* among patients. In this work, *R*_0_ is set to be approximately 0.57 based on a value provided by preceding studies (*R*_0_∼0.55−1.99) [16, 29]. Without admissions of colonized and symptomatic patients, *C. difficile* would die out from the patient population. According to the setting, transmission coefficients are approximated when additional control measures for colonized and symptomatic patients are not included (*σ*=*ε*=1) and it is more likely that the coefficients already take into account certain control policies for preventing the spread of microorganisms in general patients.

In general, colonized or symptomatic patients are often admitted to a hospital unit. Hence, we consequently investigate the prevalence of *C. difficile* when there are admissions of colonized and symptomatic patients. Based on our baseline result, the prevalence of colonized patients is approximately 26% which is quite close to the 10–25% range of colonization of *C. difficile* in hospitals in preceding studies [31, 38]. From the results, we obtain the acquisition rate of *C. difficile* in the hospital at 16% which is in agreement with the range 4–21% of *C. difficile* acquisition rates in some previous studies [27]. Moreover, we demonstrate that control strategies and admissions of colonized and symptomatic patients play important roles in determining the prevalence and the persistence of *C. difficile* in patients. Applying additional control measures to both colonized and symptomatic patients may help reduce the prevalence of *C. difficile*. Our results also suggest more stringent control policies for colonized and asymptomatic patients to obtain the lower prevalence of *C. difficile*. In addition, our results highlight a greater impact from admissions of colonized and symptomatic patients even though stringent control policies are applied.

Furthermore, based on our stochastic results, the prevalence of *C. difficile* infection fluctuates over time which is relatively in agreement with infrequent occurrences of *C. difficile* infection in other preceding works [27]. Our results suggest the tendency of *C. difficile* colonization and infection to be temporarily driven out when stringent control policies are applied to colonized and symptomatic patients. However, when admission rates of colonized and symptomatic patients are high, the absence of *C. difficile* colonization or infection from the hospital unit is less frequent. Moreover, our stochastic results are in agreement with the deterministic results to underline how the presence of colonized and symptomatic patients at admission affects the prevalence of *C. difficile* and to promote strict control policies to be applied to colonized or asymptomatic patients.

An important limitation of this study is from our parameter setting as there is no single study providing all the parameter values used in the model. Note that here parameters are sourced from multiple settings. Another limitation is the assumption that exposure to *C. difficile* is proportional to the presence of colonized and symptomatic patients without taking into account other possible sources or reservoirs from health-care workers or visitors. A further limitation of our work is from our stochastic results. Due to the very long execution time, only 800 realizations are used to calculate the absent time and the absent number.

Finally, we believe that our findings may help researchers, practitioners and public health to gain insights into how control strategies and admissions of colonized and symptomatic patients affect the prevalence and the persistence of *C. difficile* and may perhaps help suggest control strategies to reduce the spread of *C. difficile* in hospital settings. Future directions of this work would be to extend the model to include recurrent CDI.

## Abbreviations

*C. difficile*: *Clostridium difficile*
CDI: *Clostridium difficile* infection
IgA: Immunoglobulin A
IgG: Immunoglobulin G
